# Supplementary material for: Molecular Epidemiology of tet(A)-v1-Positive Carbapenem-Resistant Klebsiella pneumoniae in Pediatric Patients in a Chinese Hospital
Source: Antibiotics (Basel). 2025 Aug 22;14(9):852. doi: 10.3390/antibiotics14090852 (PMC12466384; doi:10.3390/antibiotics14090852)
Supplement: Supplementary file 1 [file antibiotics-14-00852-s001.zip › antibiotics-3798919-supplementary-proofed.pdf]

Supplementary Materials

Table S2. Results of antimicrobial susceptibility testing and conjugation assay

Strains	Minimum Inhibitory Concentration (μg/mL)																		Recipients	Conjugation rates
	AMK	AMP	SAM	ATM	SXT	CIP	TZP	GEN	FEP	CRO	CAZ	CTT	TOB	IPM	LVX	TCY	TGC	POL		
CRESXJ8	≤2	≥32	≥32	≤1	≥320	1	64	8	2	≥64	≥64	≤4	≥16	≥16	1	≥16	2	≤0.5	*E. coli* EC600	7.75×10^{-8}
CRESXJ14	≤2	≥32	≥32	≤1	≤20	≤0.25	8	≤1	16	≥32	≥64	≥64	≤1	≥16	≤0.12	≥16	2	1	*E. coli* EC600	-
CRESXJ15	≤2	≥32	≥32	≤1	≥320	1	8	≤1	16	≥64	≥64	≥64	≤1	≥16	1	≥16	2	≤0.5	*E. coli* EC600	6.46×10^{-4}
CRESXJ16	≤2	≥32	≥32	≤1	≤20	1	≤4	≤1	8	≤1	≥64	≥64	≤1	2	1	≥16	2	≤0.5	*E. coli* EC600	1.96×10^{-4}
CRESXJ21	≤2	≥32	≥32	≤1	≥320	1	8	≤1	16	≥64	≥64	≥64	≤1	≥16	1	≥16	2	≤0.5	*E. coli* EC600	4.47×10^{-5}
CRESXJ22	≤2	≥32	≥32	≤1	≥320	1	8	≤1	16	≥64	≥64	≥64	≤1	≥16	1	≥16	2	≤0.5	*E. coli* EC600	2.11×10^{-6}
CRESXJ28	≤2	≥32	≥32	≤1	≤20	≤0.25	8	≤1	16	≥64	≥64	≥64	≤1	≥16	≤0.12	≥16	2	≤0.5	*E. coli* EC600	-
CRESXJ29	≤2	≥32	≥32	≤1	≥320	≤0.25	8	≤1	16	≥64	≥64	≥64	≤1	≥16	≤0.12	≥16	2	1	*E. coli* EC600	7.05×10^{-4}
CRESXJ31	≤2	≥32	≥32	≤1	≥320	1	8	≤1	16	≥64	≥64	≥64	≤1	≥16	1	≥16	2	≤0.5	*E. coli* EC600	6.09×10^{-4}
CRESXJ34	≤2	≥32	≥32	≤1	≤20	≤0.25	8	≤1	16	≥64	≥64	≥64	≤1	≥16	≤0.12	≥16	2	≤0.5	*E. coli* EC600	2.58×10^{-8}
CRESXJ35	≤2	≥32	≥32	≤1	≥320	≤0.25	≥128	≥16	16	≥64	≥64	≥64	8	≥16	1	≥16	2	≤0.5	*E. coli* EC600	-
CRESXJ37	≤2	≥32	≥32	≤1	≤20	≤0.25	8	≤1	16	≥64	≥64	≥64	≤1	≥16	≤0.12	≥16	2	2	*E. coli* J53	1.80×10^{-7}
CRESXJ39	≤2	≥32	≥32	≤1	≤20	≤0.25	8	≤1	4	≥64	≥64	≥64	≤1	≥16	≤0.12	≥16	2	≤0.5	*E. coli* EC600	2.58×10^{-8}
CRESXJ43	≤2	≥32	≥32	≤1	≤20	≤0.25	8	≤1	16	≥64	≥64	≥64	≤1	≥16	≤0.12	≥16	2	≤0.5	*E. coli* EC600	7.75×10^{-8}
CRESXJ47	≤2	≥32	≥32	16	≥320	≥4	≥128	≤1	32	≥64	≥64	≥64	≤1	≥16	4	≥16	≥8	≤0.5	*E. coli* EC600	2.58×10^{-8}
CRESXJ49	≤2	≥32	≥32	≤1	≥320	2	≥128	≤1	16	≥64	≥64	≥64	≤1	≥16	4	≥16	≥8	2	*E. coli* EC600	5.16×10^{-8}
CRESXJ50	≤2	≥32	≥32	16	≥320	≥4	≥128	≤1	16	≥64	≥64	TRM	≤1	≥16	4	≥16	≥8	2	*E. coli* EC600	1.96×10^{-6}
CRESXJ51	≤2	≥32	≥32	≤1	≥320	2	≥128	≤1	16	≥64	≥64	≥64	≤1	≥16	4	≥16	≥8	2	*E. coli* EC600	1.29×10^{-7}
CRESXJ53	≤2	≥32	≥32	16	≥320	≥4	≥128	≤1	16	≥64	≥64	≥64	≤1	≥16	4	≥16	2	≤0.5	*E. coli* EC600	5.16×10^{-8}
CRESXJ57	≤2	≥32	≥32	16	≥320	≥4	≥128	≤1	≥32	≥64	≥64	≥64	≤1	≥16	4	≥16	2	2	*E. coli* EC600	1.29×10^{-7}
CRESXJ58	≤2	≥32	≥32	≤1	≥320	2	8	≤1	16	≥64	≥64	≥64	≤1	≥16	4	≥16	≥8	≤0.5	*E. coli* EC600	2.11×10^{-3}
CRESXJ65	≤2	≥32	≥32	≤1	≥320	1	8	≤1	16	≥64	≥64	≥64	≤1	≥16	1	≥16	2	≤0.5	*E. coli* EC600	3.48×10^{-3}

BSIKP_26	4	≥32	≥32	4	≥320	≥4	64	≤1	16	≥64	≥64	≥64	≥16	≥16	≥8	≥16	1	1	*E. coli* EC600	6.45×10^{-7}
BSIKP_28	≤2	≥32	≥32	2	≥320	1	≥128	≤1	16	≥64	≥64	≥64	≤1	≥16	1	≥16	0.5	1	*E. coli* EC600	5.68×10^{-5}
BSIKP_59	≤2	≥32	≥32	2	≥320	1	≥128	≤1	16	≥64	≥64	≥64	≤1	≥16	1	≥16	0.5	1	*E. coli* EC600	1.75×10^{-6}
BSIKP_67	≤2	≥32	≥32	≤1	≥320	1	8	≤1	≥64	≥64	≥64	≥64	≤1	8	1	≥16	1	1	*E. coli* EC600	1.32×10^{-3}
BSIKP_68	≤2	≥32	≥32	2	≥320	1	8	≤1	≥64	≥64	≥64	≥64	≤1	≥16	1	≥16	1	1	*E. coli* EC600	9.19×10^{-4}
BSIKP_69	≥64	≥32	≥32	≥64	≥320	≥4	≥128	≥16	≥64	≥64	≥64	≥64	≥16	≥16	≥8	≥16	1	1	*E. coli* J53	-
BSIKP_70	≤2	≥32	≥32	≤1	≥320	2	≥128	≤1	≥64	≥64	≥64	≥64	≤1	≥16	2	≥16	2	2	*E. coli* EC600	2.58×10^{-7}
BSIKP_71	≤2	≥32	≥32	≤1	≤20	≤0.25	8	≤1	4	≥64	≥64	≥64	≤1	4	≤0.25	≥16	0.5	1	*E. coli* EC600	-
BSIKP_111	32	≥32	≥32	16	≥320	1	≥128	≤1	≥64	≥64	≥64	≥64	≥16	≥16	1	≥16	1	1	*E. coli* EC600	7.75×10^{-8}
BSIKP_114	16	≥32	≥32	≥64	≥320	2	≥128	≤1	≥64	≥64	≥64	≥64	≥16	≥16	1	≥16	1	1	*E. coli* EC600	7.75×10^{-8}
BSIKP_115	32	≥32	≥32	4	≥320	2	≥128	4	≥64	≥64	≥64	≥64	≥16	≥16	2	≥16	1	1	*E. coli* EC600	5.16×10^{-8}
BSIKP_E9	≤2	≥32	≥32	16	≥320	2	≥128	≤1	≥64	≥64	≥64	≥64	≤1	≥16	2	≥16	1	2	*E. coli* EC600	-
GJY_G3	4	≥32	≥32	4	≥320	≥4	≥128	≤1	≥64	≥64	≥64	≥64	≥16	≥16	1	≥16	1	2	*E. coli* EC600	1.26×10^{-6}
GJY_G4	≥64	≥32	≥32	4	≥320	2	≥128	4	≥64	≥64	≥64	≥64	≥16	≥16	2	≥16	1	2	*E. coli* EC600	8.01×10^{-6}
GJY_G6	16	≥32	≥32	≥64	≥320	2	≥128	≤1	≥64	≥64	≥64	≥64	≥16	≥16	1	≥16	0.5	≥4	*E. coli* EC600	1.03×10^{-7}
HLLCRE64-16	≤2	≥32	≥32	≤1	≤20	≤0.25	8	≤1	2	≥64	≥64	≥64	≤1	4	≤0.25	≥16	0.25	2	*E. coli* EC600	7.75×10^{-8}
HLLCRE64-17	≤2	≥32	≥32	≤1	≥320	1	8	≤1	≥64	≥64	≥64	≥64	≤1	8	1	≥16	1	2	*E. coli* EC600	1.05×10^{-3}
HLLCRE64-19	≤2	≥32	≥32	≤1	≥320	1	8	≤1	≥64	≥64	≥64	≥64	≤1	8	1	≥16	0.5	2	*E. coli* EC600	1.73×10^{-4}
HLLCRE64-22	≤2	≥32	≥32	≤1	≤20	≤0.25	8	≤1	4	≥64	≥64	≥64	≤1	4	≤0.25	≥16	0.25	2	*E. coli* EC600	2.58×10^{-8}
HLLCRE64-23	≤2	≥32	≥32	≤1	≥320	0.5	≥128	≥16	≥64	≥64	≥64	≥64	8	≥16	1	≥16	0.25	2	*E. coli* J53	-
HLLCRE64-25	≤2	≥32	≥32	≤1	≤20	≤0.25	8	≤1	2	≥64	≥64	≥64	≤1	4	≤0.25	≥16	0.5	2	*E. coli* EC600	1.55×10^{-7}
HLLCRE64-27	≤2	≥32	≥32	≤1	≤20	≤0.25	8	≤1	2	≥64	≥64	≥64	≤1	4	≤0.25	≥16	0.25	2	*E. coli* EC600	-
HLLCRE64-31	≤2	≥32	≥32	≤1	≤20	≤0.25	8	≤1	2	≥64	≥64	≥64	≤1	4	≤0.25	≥16	0.25	2	*E. coli* EC600	-
HLLCRE64-36	≤2	≥32	16	16	≤20	≤0.25	≤4	≤1	≤1	8	16	≤4	≤1	≤1	≤0.25	≥16	≤0.125	2	*E. coli* EC600	-
HLLCRE64-37	≤2	≥32	≥32	≤1	≥320	2	≥128	≤1	≥64	≥64	≥64	≥64	≤1	≥16	2	≥16	1	2	*E. coli* EC600	-
HLLCRE64-38	≤2	≥32	≥32	16	≥320	≥4	≥128	≤1	≥64	≥64	≥64	≥64	≤1	≥16	4	≥16	1	2	*E. coli* EC600	-
HLLCRE64-39	≤2	≥32	≥32	16	≥320	2	≥128	≤1	≥64	≥64	≥64	≥64	≤1	≥16	2	≥16	0.5	2	*E. coli* EC600	-
HLLCRE64-4	≤2	≥32	≥32	≤1	≥320	1	8	≤1	≥64	≥64	≥64	≥64	≤1	2	1	≥16	≤0.125	2	*E. coli* EC600	3.10×10^{-4}

HLLCRE64-41	≤2	≥32	≥32	16	≥320	≥4	≥128	≤1	≥64	≥64	≥64	≥64	≤1	≥16	4	≥16	1	2	*E. coli* EC600	5.42×10^{-7}
HLLCRE64-45	≤2	≥32	≥32	≥64	≥320	≥4	≥128	≤1	≥64	≥64	≥64	≥64	≤1	≥16	2	≥16	0.5	2	*E. coli* EC600	1.29×10^{-7}
HLLCRE64-5	≤2	≥32	≥32	≤1	≥320	1	8	≤1	≥64	≥64	≥64	≥64	≤1	8	1	≥16	0.5	≤0.5	*E. coli* EC600	1.29×10^{-6}
HLLCRE64-50	≤2	≥32	≥32	≤1	≤20	≤0.25	8	≤1	2	16	≥64	≥64	≤1	2	≤0.25	≥16	≤0.125	2	*E. coli* EC600	-
HLLCRE64-52	≤2	≥32	≥32	≤1	≥320	1	≤4	≤1	2	≥64	≥64	≥64	≤1	≤1	1	≥16	0.5	2	*E. coli* EC600	4.13×10^{-6}
HLLCRE64-53	≤2	≥32	≥32	≤1	≤20	1	8	≤1	8	≥64	≥64	≥64	≤1	≥16	1	≥16	1	2	*E. coli* EC600	-
HLLCRE64-54	≤2	≥32	≥32	≤1	≤20	1	32	≤1	≥64	≥64	≥64	≥64	≤1	8	1	≥16	2	2	*E. coli* EC600	-
HLLCRE64-55	≤2	≥32	≥32	4	≥320	0.5	≥128	≥16	≥64	≥64	≥64	≥64	8	≥16	1	≥16	0.25	2	*E. coli* EC600	-
HLLCRE64-58	≤2	≥32	≥32	≤1	≤20	1	8	≤1	8	≥64	≥64	≥64	≤1	≥16	1	≥16	0.25	≥4	*E. coli* EC600	-
HLLCRE64-61	≤2	≥32	≥32	≤1	≤20	1	8	≤1	16	≥64	≥64	≥64	≤1	8	1	≥16	0.25	≥4	*E. coli* EC600	7.75×10^{-7}
HLLCRE64-62	≤2	≥32	≥32	8	≤20	≥4	≥128	≤1	≥64	≥64	≥64	≥64	≤1	≥16	≥8	≥16	0.25	2	*E. coli* EC600	1.55×10^{-6}
terRKP_13	4	≥32	≥32	≥64	≥320	≥4	64	≤1	≥64	≥64	≥64	≤4	≥16	≤1	≥8	≥16	0.25	2	*E. coli* EC600	-
terRKP_228	≤2	≥32	≥32	≤1	≥320	0.5	≥128	≥16	≥64	≥64	≥64	≥64	≥16	≥16	1	≥16	4	1	*E. coli* EC600	2.06×10^{-6}
terRKP_23	≥64	≥32	≥32	4	≥320	2	≥128	4	≥64	≥64	≥64	≥64	≥16	≥16	2	≥16	2	1	*E. coli* EC600	7.75×10^{-7}
terRKP_25	16	≥32	≥32	4	≥320	≥4	64	≤1	16	≥64	≥64	≥64	8	≥16	4	≥16	4	1	*E. coli* J53	1.03×10^{-5}
terRKP_428	≤2	≥32	≥32	≤1	≥320	1	8	≤1	≥64	≥64	≥64	≥64	≤1	2	1	≥16	2	1	*E. coli* J53	-
terRKP_460	≤2	≥32	≥32	≤1	≥320	1	8	≤1	≥64	≥64	≥64	≥64	≤1	8	1	≥16	1	1	*E. coli* EC600	1.78×10^{-6}
terRKP_467	≤2	≥32	≥32	≤1	≥320	1	8	≤1	≥64	≥64	≥64	≥64	≤1	8	1	≥16	0.5	1	*E. coli* EC600	5.42×10^{-5}
terRKP_473	≤2	≥32	≥32	≤1	≥320	0.5	≥128	≥16	≥64	≥64	≥64	≥64	8	≥16	1	≥16	0.5	1	*E. coli* J53	-
terRKP_47	≤2	≥32	≥32	≤1	≥320	1	≥128	≥16	≥64	≥64	≥64	≥64	8	≥16	1	≥16	1	1	*E. coli* EC600	-
terRKP_612	≤2	≥32	≥32	2	≥320	1	≥128	≤1	≥64	≥64	≥64	≥64	≤1	8	≥8	≥16	0.5	1	*E. coli* EC600	2.06×10^{-7}
terRKP_76	≥64	≥32	≥32	≥64	≥320	≥4	≥128	≥16	≥32	≥64	≥64	≥64	≥16	≥16	≥8	≥16	≥8	1	*E. coli* EC600	1.03×10^{-5}
terRKP_870	≤2	≤2	≤2	2	≥320	1	≥128	≤1	8	≤1	≥64	≥64	≤1	8	1	≥16	2	2	*E. coli* EC600	-

Abbreviations: AMK, Amikacin; AMP, Ampicillin; SAM, Ampicillin/Sulbactam; ATM, Aztreonam; SXT, Trimethoprim/Sulfamethoxazole; CIP, Ciprofloxacin; TZP, piperacillin/tazobactam; GEN, Gentamicin; FEP, Cefepime; CRO, Ceftriaxone; CAZ, Ceftazidime; CTT, Cefotetan; TOB, Tobramycin; IPM, Imipenem; LVX, Levofloxacin; TCY, Tetracycline; TGC, Tigecycline; POL, Polymyxin B.

-, non-conjugative.

Table S3. Antimicrobial susceptibility of *tet*(A)-v1-positive CRKP from pediatric patients.

Categories of. Antibiotics	Antimicrobial agent	Breakpoints (μg/mL) (Susceptibility/Resistance)	MIC_{50} (μg/mL)	MIC_{90} (μg/mL)	S%	I%	R%
β-lactams	Ampicillin	≤8 / ≥32	≥32	≥32	1.37	0	98.63
	Ampicillin/Sulbactam	≤8/4/ ≥16/8	≥32	≥32	1.37	1.37	97.26
	Piperacillin/tazobactam	≤8/4 / ≥32/2	64	≥128	47.96	0	52.04
	Aztreonam	≤4 / 16	≤1	16	76.71	1.37	21.92
	Cefepime	≤2 / ≥16	16	≥64	20.55	0	79.45
	Ceftriaxone	≤1 / ≥4	≥64	≥64	2.74	0	97.26
	Ceftazidime	≤2 / ≥16	≥64	≥64	0	0	100
	Cefotetan	≤16 / ≥64	≥64	≥64	4.11	0	95.89
	Imipenem	≤1 / ≥4	≥16	≥16	4.11	5.48	90.41
Sulfonamides	Trimethoprim/Sulfamethoxazole	≤2/32 / ≥4/76	≥320	≥320	26.03	0	73.97
Polymyxins	Polymyxin B	-/ ≥4	1	2	95.89	0	4.11
Aminoglycosides	Amikacin	≤16 / ≥64	≤2	16	87.67	0	12.33
	Gentamicin	≤4 / ≥16	≤1	≥16	83.56	4.11	12.33
	Tobramycin	≤4 / ≥16	≤1	≥16	73.97	0	26.03
Quinolone	Ciprofloxacin	≤1 / ≥4	1	≥4	21.92	5.48	72.60
	Levofloxacin	≤0.5/ ≥8	1	4	20.55	46.57	32.88
Tetracycline	Tetracycline	≤4 / ≥16	≥16	≥16	0	0	100
Glycylcycline	Tigecycline	≤2 / ≥8	1	4	89.04	2.74	8.22

Abbreviations: MIC_{50} and MIC_{90} represent the MIC required to inhibit the growth of 50% and 90% of the tested bacteria, respectively; S, susceptibility; I, intermediate; R, resistance; "-", not applicable.

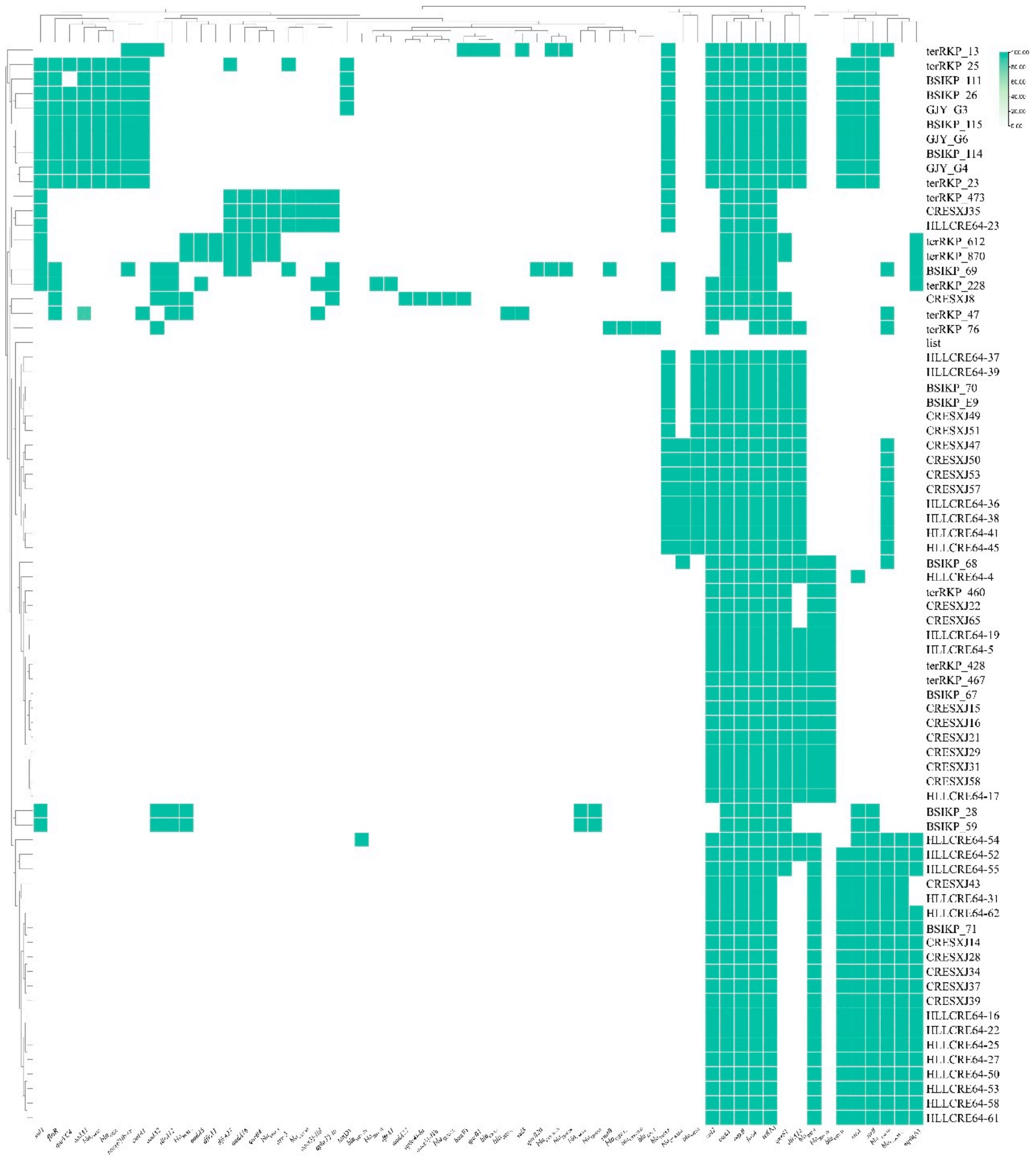

Figure S1. Resistance gene heatmap. The resistance gene heatmap provides a visual representation of the distribution of various resistance genes across the 73 CRKP isolates. Each row corresponds to a different isolate, and each column represents a specific resistance gene. Green indicates the presence of a resistance gene, while white indicates its absence. The dendrogram on the left side of the heatmap shows the hierarchical clustering of the isolates, highlighting the phylogenetic relationships.

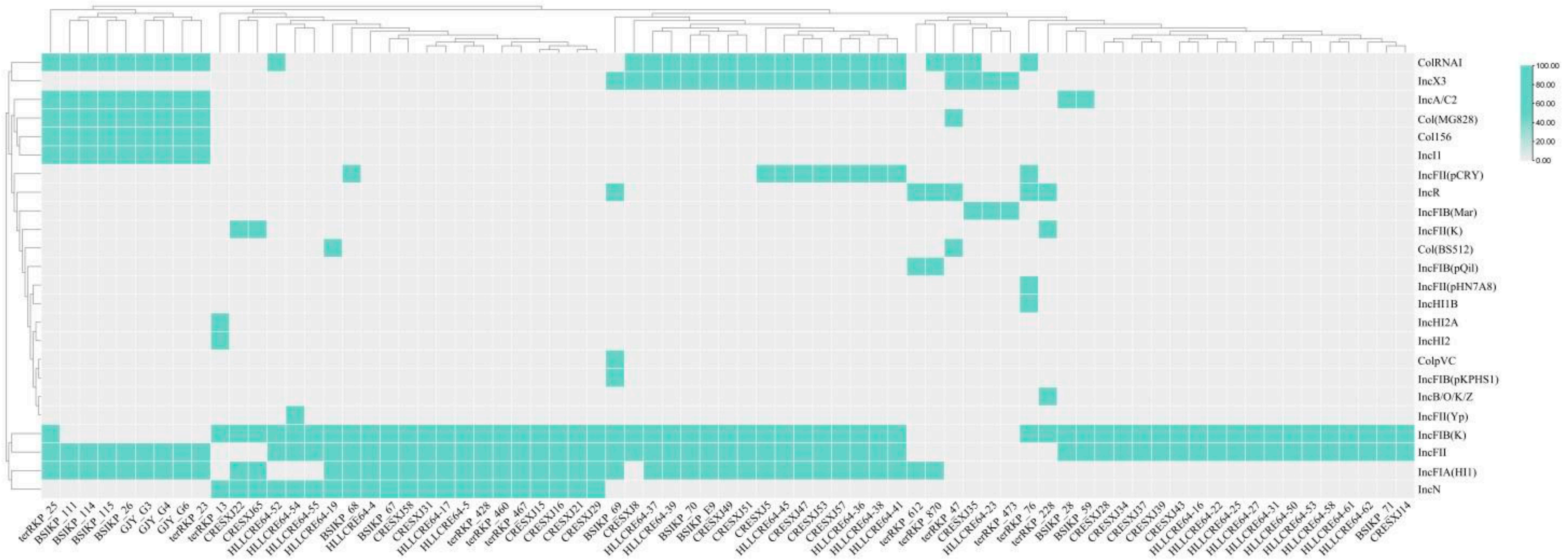

Figure S2. Plasmid heatmap. The plasmid heatmap illustrates the distribution of plasmid types among the 73 CRKP isolates, organized by their sequence types (STs). Each row corresponds to an individual isolate grouped by ST classification, while columns represent specific plasmid types or resistance genes. A color-coded system (green for presence, gray for absence/inconclusive data) denotes plasmid carriage patterns. The left-sided dendrogram demonstrates ST-based clustering, revealing potential epidemiological linkages among isolates of the same ST lineage. Notably, this ST-oriented visualization enables direct correlation between plasmid profiles and bacterial clonal backgrounds.

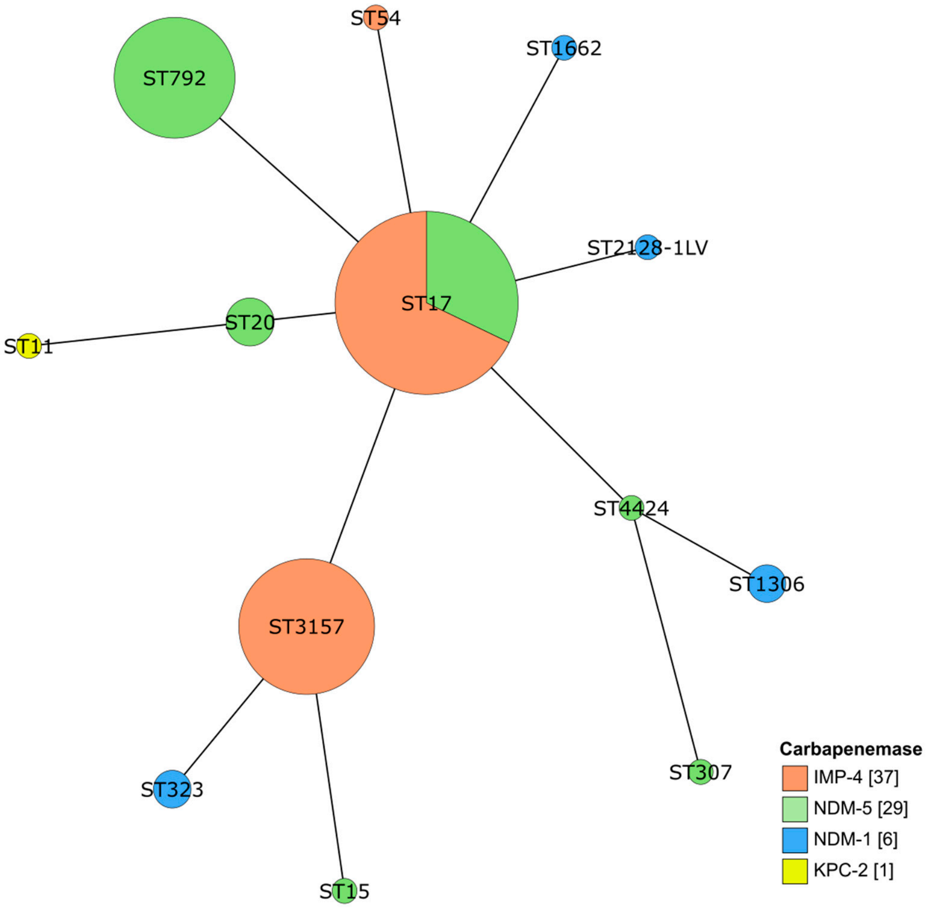

Figure S3. The Grapelree Tree provides a comprehensive visualization of the phylogenetic relationships among the 73 CRKP isolates, integrating additional data such as STs and carbapenem resistance genes. Orange, green, blue and yellow represent the carbapenem resistance genes *bla*$_{IMP4}$, *bla*$_{NDM-5}$, *bla*$_{NDM-1}$, and *bla*$_{KPC-2}$, respectively.

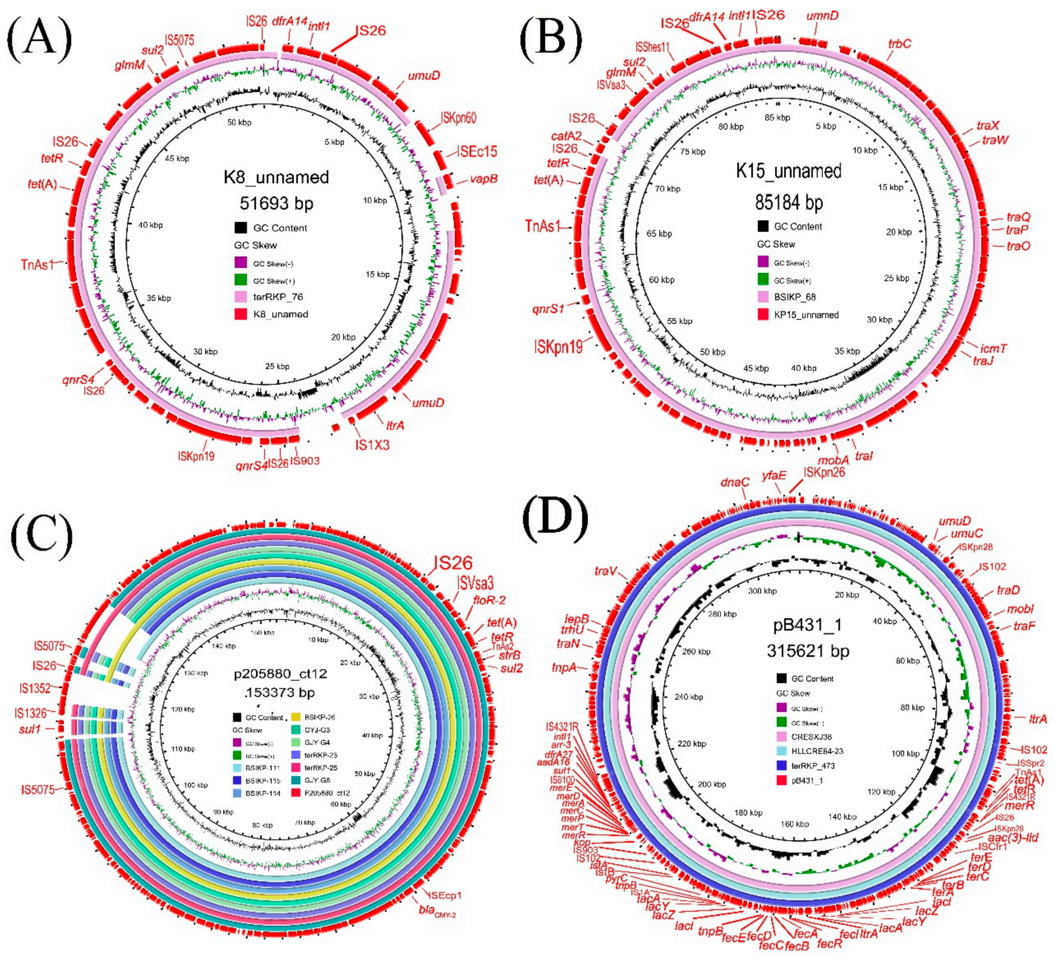

Figure S4. Circular plasmid map. **(A)** Circular plasmid map of K8_unnamed. The plasmids from innermost to outermost circles are pterRKP_76 (this study) and K8_unnamed (GenBank accession: CP126620), respectively. Antimicrobial resistance genes and mobile genetic elements are labeled in the outermost circle. **(B)** Circular plasmid map of KP15_unnamed. The plasmids from innermost to outermost circles are pBSIKP_68 (this study) and K15_unnamed (GenBank accession: CP146604), respectively. Antimicrobial resistance genes and mobile genetic elements are labeled in the outermost circle. **(C)** Circular plasmid map of p205880-ct12. The plasmids from innermost to outermost circles are pBSIKP_111, pBSIKP_115, pBSIKP_114, pBSIKP_26, pGJY_G3, pGJY_G4, pterRKP_23, pterRKP_25, pGJY_G6, (this study), and p205880-ct12 (GenBank accession: MF344573), respectively. Antimicrobial resistance genes and mobile genetic elements are labeled in the outermost circle. **(D)** Circular plasmid map of pB431_1. The plasmids from innermost to outermost circles are pCRESXJ_38, pHLLCRE64-23, pterRKP_473 (this study), and pB431_1 (GenBank accession: CP110662), respectively. Antimicrobial resistance genes and mobile genetic elements are labeled in the outermost circle.

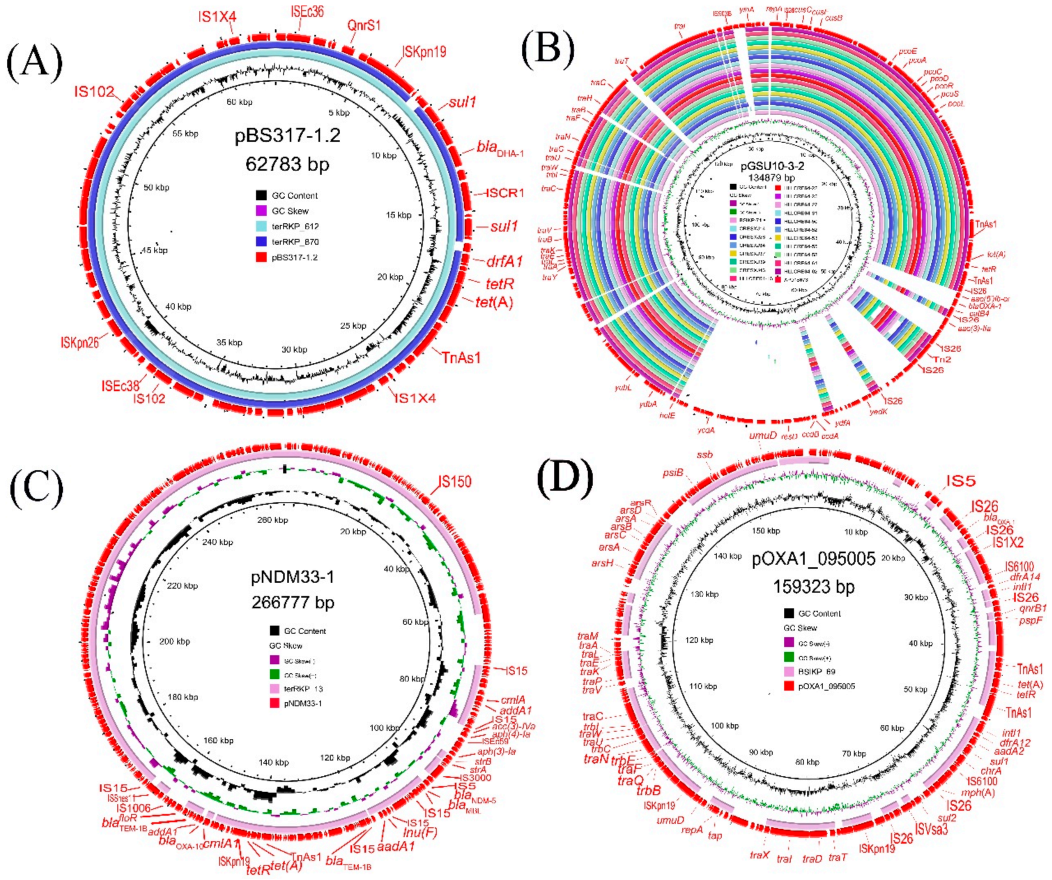

Figure S5. Circular plasmid map. **(A)** Circular plasmid map of pBS317-1.2. The plasmids from innermost to outermost circles are pterRKP_812, pterRKP_870, (this study), and pBS317-1.2 (GenBank accession: CP063938), respectively. Antimicrobial resistance genes and mobile genetic elements are labeled in the outermost circle. **(B)** Circular plasmid map of pGSU10-3-2. The plasmids from innermost to outermost circles are pBSIKP_71, pCRESXJ14, pCRESXJ28, pCRESXJ34, pCRESXJ37, pCRESXJ39, pCRESXJ43, pHLLCRE64-16, pHLLCRE64-22, pHLLCRE64-25, pHLLCRE64-27, pHLLCRE64-31, pHLLCRE64-50, pHLLCRE64-52, pHLLCRE64-53, pHLLCRE64-55, pHLLCRE64-58, pHLLCRE64-61, pHLLCRE64-62 (this study), and pGSU10-3-2 (GenBank accession: AP018673), respectively. Antimicrobial resistance genes and mobile genetic elements are labeled in the outermost circle. **(C)** Circular plasmid map of pNDM33-1. The plasmids from innermost to outermost circles are pterRKP_13 (this study) and pNDM33-1 (GenBank accession: MN915011), respectively. Antimicrobial resistance genes and mobile genetic elements are labeled in the outermost circle. **(D)** Circular plasmid map of pOXA1-09005. The plasmids from innermost to outermost circles are pBSIKP_69 (this study), and pOXA1-09005 (GenBank accession: CP043380), respectively. Antimicrobial resistance genes and mobile genetic elements are labeled in the outermost circle.

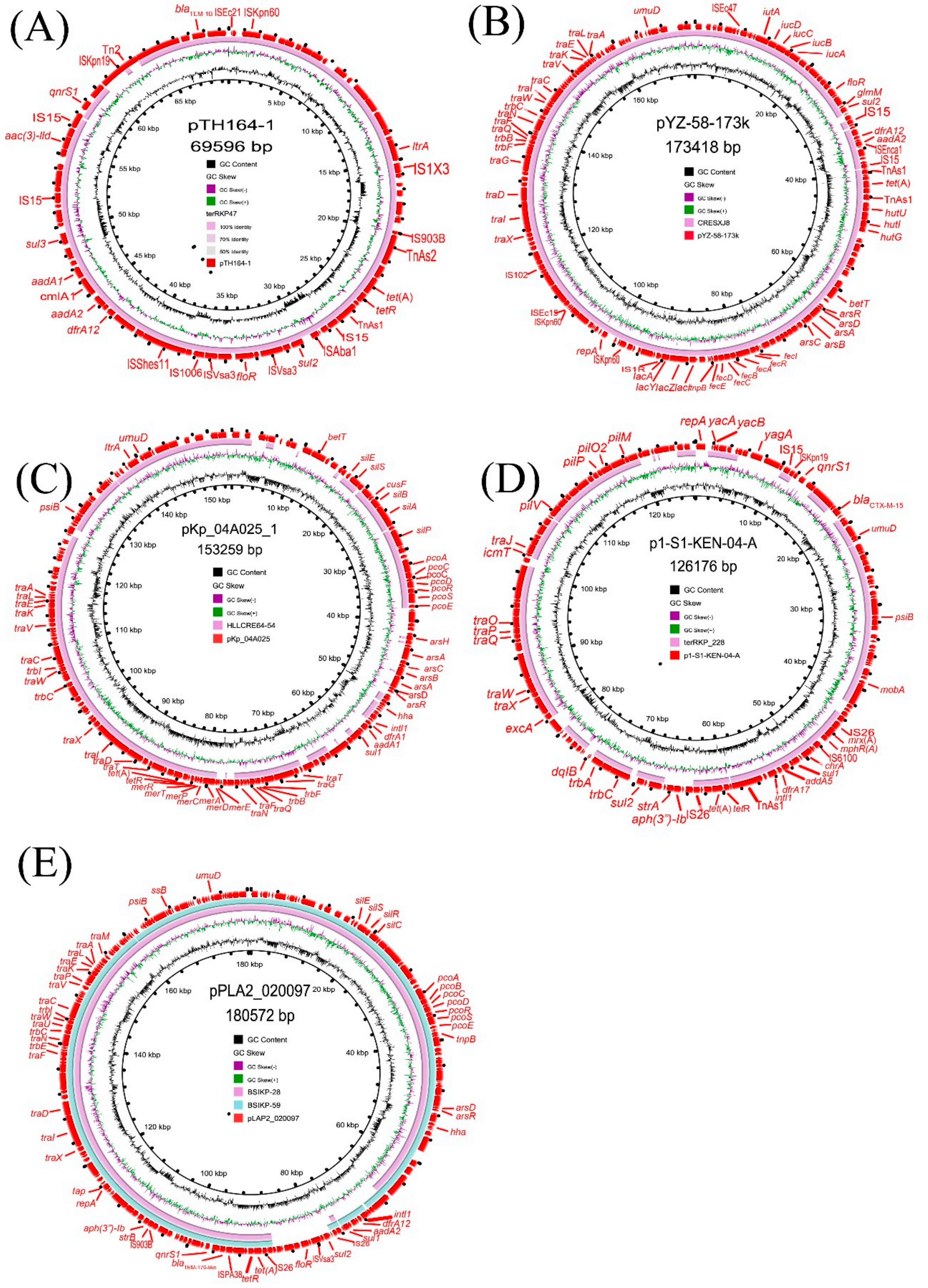

Figure S6. Circular plasmid map. **(A)** Circular plasmid map of pTH164-1. The plasmids from innermost to outermost circles are pterRKP_47 (this study) and pTH164-1 (GenBank accession: CP035212), respectively. Antimicrobial resistance genes and mobile genetic elements are labeled in the outermost circle. **(B)** Circular plasmid map of pYZ_58-173k. The plasmids from innermost to outermost circles are pCRESXJ_8 (this study) and pYZ_58-173k (GenBank accession: CP109774), respectively.

Antimicrobial resistance genes and mobile genetic elements are labeled in the outermost circle. **(C)** Circular plasmid map of pKp_04A025_1. The plasmids from innermost to outermost circles are pHLLCRE64-54 (this study) and pKp_04A025_1 (GenBank accession: CP084872), respectively. Antimicrobial resistance genes and mobile genetic elements are labeled in the outermost circle. **(D)** Circular plasmid map of p1-S1-KEN-04-A. The plasmids from innermost to outermost circles are pterRKP_228 (this study) and p1-S1-KEN-04-A (GenBank accession: CP145691), respectively. Antimicrobial resistance genes and mobile genetic elements are labeled in the outermost circle. **(E)** Circular plasmid map of pPLA2_020097. The plasmids from innermost to outermost circles are pBSIKP_28, pBSIKP_59 (this study) and pPLA2_020097 (GenBank accession: CP04335), respectively. Antimicrobial resistance genes and mobile genetic elements are labeled in the outermost circle.

Table S4. Number of SNP differences in ST17

snp-dists 0.8.2	BSIKP 111	BSIKP 114	BSIKP 115	BSIKP 26	GJY-G3	GJY-G4	GJY-G6	terRKP _23	terRKP _25	BSIK P71	HLL CRE 64-16	HLL CRE 64-22	HLL CRE 64-25	HLL CRE 64-27	HLL CRE 64-31	HLL CRE 64-50	HLL CRE 64-52	HLL CRE 64-53	HLL CRE 64-55	HLL CRE 64-58	HLL CRE 64-61	HLL CRE 64-62	CRE SXJ 14	CRE SXJ 28	CRE SXJ3 4	CRE SXJ 37	CRE SXJ 39	CRE SXJ 43
BSIKP-111	0	25	20	10	12	20	27	22	13	3291	3316	3322	3315	3322	3319	3309	3311	3311	3312	3312	3314	3311	3311	3322	3323	3321	3322	3321
BSIKP114	25	0	4	15	13	5	2	6	18	3271	3296	3302	3295	3302	3299	3289	3292	3291	3292	3292	3294	3291	3291	3302	3303	3301	3302	3301
BSIKP115	20	4	0	10	8	0	6	1	13	3267	3292	3298	3291	3298	3295	3285	3288	3287	3288	3288	3290	3287	3287	3298	3299	3297	3298	3297
BSIKP26	10	15	10	0	2	10	17	11	3	3250	3275	3281	3274	3281	3278	3268	3271	3270	3271	3271	3273	3270	3270	3281	3282	3280	3281	3280
GJY-G3	12	13	8	2	0	8	15	9	5	3260	3285	3291	3284	3291	3288	3278	3281	3280	3281	3281	3283	3280	3280	3291	3292	3290	3291	3290
GJY-G4	20	5	0	10	8	0	7	1	13	3268	3293	3299	3292	3299	3296	3286	3289	3288	3289	3289	3291	3288	3288	3299	3300	3298	3299	3298
GJY-G6	27	2	6	17	15	7	0	9	20	3275	3300	3306	3299	3306	3303	3293	3296	3295	3296	3296	3298	3295	3295	3306	3307	3305	3306	3305
terRKP_23	22	6	1	11	9	1	9	0	15	3270	3295	3301	3294	3301	3298	3288	3291	3290	3291	3291	3293	3290	3290	3301	3302	3300	3301	3300
terRKP_25	13	18	13	3	5	13	20	15	0	2136	2159	2160	2152	2160	2158	2148	2150	2150	2151	2150	2152	2150	2149	2160	2161	2160	2160	2159
BSIKP71	3291	3271	3267	3250	3260	3268	3275	3270	2136	0	1	1	1	1	1	12	14	13	12	11	13	11	11	1	1	1	1	1
HLLCRE64-16	3316	3296	3292	3275	3285	3293	3300	3295	2159	1	0	1	0	0	0	11	13	12	11	10	12	10	10	0	1	0	0	0
HLLCRE64-22	3322	3302	3298	3281	3291	3299	3306	3301	2160	1	1	0	1	1	1	12	14	13	12	11	13	11	11	1	0	1	1	1
HLLCRE64-25	3315	3295	3291	3274	3284	3292	3299	3294	2152	1	0	1	0	0	0	11	13	12	11	10	12	10	10	0	1	0	0	0
HLLCRE64-27	3322	3302	3298	3281	3291	3299	3306	3301	2160	1	0	1	0	0	0	11	13	12	11	10	12	10	10	0	1	0	0	0
HLLCRE64-31	3319	3299	3295	3278	3288	3296	3303	3298	2158	1	0	1	0	0	0	11	13	12	11	10	12	10	10	0	1	0	0	0
HLLCRE64-50	3309	3289	3285	3268	3278	3286	3293	3288	2148	12	11	12	11	11	11	0	4	3	2	1	3	1	1	11	12	11	11	11
HLLCRE64-52	3311	3292	3288	3271	3281	3289	3296	3291	2150	14	13	14	13	13	13	4	0	3	4	3	5	3	3	13	14	13	13	13
HLLCRE64-53	3311	3291	3287	3270	3280	3288	3295	3290	2150	13	12	13	12	12	12	3	3	0	3	2	4	2	2	12	13	12	12	12
HLLCRE64-55	3312	3292	3288	3271	3281	3289	3296	3291	2151	12	11	12	11	11	11	2	4	3	0	1	3	1	1	11	12	11	11	11

HLLCRE64-58	3312	3292	3288	3271	3281	3289	3296	3291	2150	11	10	11	10	10	10	1	3	2	1	0	2	0	0	10	11	10	10	10
HLLCRE64-61	3314	3294	3290	3273	3283	3291	3298	3293	2152	13	12	13	12	12	12	3	5	4	3	2	0	2	2	12	13	12	12	12
HLLCRE64-62	3311	3291	3287	3270	3280	3288	3295	3290	2150	11	10	11	10	10	10	1	3	2	1	0	2	0	0	10	11	10	10	10
CRESXJ14	3311	3291	3287	3270	3280	3288	3295	3290	2149	11	10	11	10	10	10	1	3	2	1	0	2	0	0	10	11	10	10	10
CRESXJ28	3322	3302	3298	3281	3291	3299	3306	3301	2160	1	0	1	0	0	0	11	13	12	11	10	12	10	10	0	1	0	0	0
CRESXJ34	3323	3303	3299	3282	3292	3300	3307	3302	2161	1	1	0	1	1	1	12	14	13	12	11	13	11	11	1	0	1	1	1
CRESXJ37	3321	3301	3297	3280	3290	3298	3305	3300	2160	1	0	1	0	0	0	11	13	12	11	10	12	10	10	0	1	0	0	0
CRESXJ39	3322	3302	3298	3281	3291	3299	3306	3301	2160	1	0	1	0	0	0	11	13	12	11	10	12	10	10	0	1	0	0	0
CRESXJ43	3321	3301	3297	3280	3290	3298	3305	3300	2159	1	0	1	0	0	0	11	13	12	11	10	12	10	10	0	1	0	0	0

Table S5. Number of SNP differences in ST20

snp-dists 0.8.2	HLLCRE64-23	CRESXJ35	terRKP_473
HLLCRE64-23	0	11	0
CRESXJ35	11	0	11
terRKP_473	0	11	0

Table S6. Number of SNP differences in ST323

snp-dists 0.8.2	BSIKP_59	BSIKP_28
BSIKP_59	0	13
BSIKP_28	13	0

Table S7. Number of SNP differences in ST1306

snp-dists 0.8.2	terRKP_612	terRKP_870
terRKP_612	0	30329
terRKP_870	30329	0

Table S8. Number of SNP differences in ST792

snp-dists 0.8.2	BSIKP_E9	HLLCRE64-36	HLLCRE64-37	HLLCRE64-38	HLLCRE64-39	HLLCRE64-41	HLLCRE64-45	BSIKP-70	SXJ47	SXJ49	SXJ50	SXJ51	SXJ53	SXJ57
BSIKP_E9	0	3	0	0	0	0	2	6	2	0	0	0	0	2
HLLCRE64-36	3	0	3	3	3	3	5	7	1	3	3	3	3	5
HLLCRE64-37	0	3	0	0	0	0	2	6	2	0	0	0	0	2
HLLCRE64-38	0	3	0	0	0	0	2	6	2	0	0	0	0	2
HLLCRE64-39	0	3	0	0	0	0	2	6	2	0	0	0	0	2
HLLCRE64-41	0	3	0	0	0	0	2	6	2	0	0	0	0	2
HLLCRE64-45	2	5	2	2	2	2	0	9	4	2	2	2	2	0
Reference	6	7	6	6	6	6	9	0	8	6	6	6	6	9
SXJ47	2	1	2	2	2	2	4	8	0	2	2	2	2	4
SXJ49	0	3	0	0	0	0	2	6	2	0	0	0	0	2
SXJ50	0	3	0	0	0	0	2	6	2	0	0	0	0	2
SXJ51	0	3	0	0	0	0	2	6	2	0	0	0	0	2
SXJ53	0	3	0	0	0	0	2	6	2	0	0	0	0	2
SXJ57	2	5	2	2	2	2	0	9	4	2	2	2	2	0

Table S9. Number of SNP differences in ST3157

snp-dists 0.8.2	BSIKP_67	BSIKP_68	SXJ58	SXJ65	HLLCRE64-17	HLLCRE64-19	HLLCRE64-4	HLLCRE64-5	SXJ15	SXJ16	SXJ21	SXJ22	SXJ29	SXJ31	terRKP_428	terRKP_460	terRKP_467
BSIKP_67	0	14	23	114	516	515	528	543	518	512	551	550	516	523	521	552	516
BSIKP_68	14	0	12	85	485	484	497	512	487	478	520	519	485	490	488	521	485
SXJ58	23	12	0	107	503	502	515	530	505	499	535	534	503	507	506	536	503
SXJ65	114	85	107	0	424	423	436	451	426	417	456	455	424	426	427	457	424
HLLCRE64-17	516	485	503	424	0	2	6	6	2	2	6	6	1	2	3	7	1
HLLCRE64-19	515	484	502	423	2	0	4	4	0	0	4	4	1	0	1	5	1
HLLCRE64-4	528	497	515	436	6	4	0	0	4	4	0	0	5	4	5	1	5
HLLCRE64-5	543	512	530	451	6	4	0	0	4	4	0	0	5	4	5	1	5
SXJ15	518	487	505	426	2	0	4	4	0	0	4	4	1	0	0	5	1
SXJ16	512	478	499	417	2	0	4	4	0	0	4	4	1	0	1	5	1
SXJ21	551	520	535	456	6	4	0	0	4	4	0	0	5	4	5	1	5
SXJ22	550	519	534	455	6	4	0	0	4	4	0	0	5	4	5	1	5
SXJ29	516	485	503	424	1	1	5	5	1	1	5	5	0	1	2	6	0
SXJ31	523	490	507	426	2	0	4	4	0	0	4	4	1	0	1	5	1
terRKP_428	521	488	506	427	3	1	5	5	0	1	5	5	2	1	0	6	2
terRKP_460	552	521	536	457	7	5	1	1	5	5	1	1	6	5	6	0	5
terRKP_467	516	485	503	424	1	1	5	5	1	1	5	5	0	1	2	5	0

In Tables S4-S9, green annotations highlight strains with >25 SNP differences compared to the reference strains.

Supplementary Results

The remaining plasmids show the following alignments and characteristics: pB431-1 (315,621 bp) aligns with ST20 (n=3) and belongs to the IncFIB. pBS317-1.2 (62,783 bp), K8_unnamed (51,693 bp), and pTH164-1 (69,596 bp) all belong to the IncR/N and align with ST1306 (n=2), ST11 (n=1), and ST1662 (n=1), respectively **(Figures S4-6)**. The plasmids pB431-1, pBS317-1.2, and pTH164-1 carry various antibiotic resistance genes as described below: pB431-1 carries *tet*(A)-v1, *acc(3)-IId, sul1*, *aadA16*, and *dfrA27* **(Figure S4B)**; pBS317-1.2 carries *tet*(A)-v1, *qnrS1*, *sul1*, *bla*$_{DHA-1}$, *dfrA1* **(Figure S5A)**; pTH164-1 carries an extensive set of resistance genes including *tet*(A)-v1, *sul2*, *floR*, *dfrA12*, *aadA2*, *cmlA1*, *aadA1*, *sul3*, *acc(3)-IId*, *qnrS1*, and *bla*$_{TEM-1B}$ **(Figure S6A)**.These genes confer resistance to different classes of antibiotics. For example, *acc(3)-IId* and *bla*$_{DHA-1}$ are associated with resistance to aminoglycosides and β-lactams, respectively, while *sul1*, *sul2*, and *sul3* confer resistance to sulfonamides. pPLA_020097 (180,572 bp), pOXA1_095005 (159323 bp), pYZ-58-173k (173418 bp), and pKp_04A025_1 (153259 bp) aligns with ST323 (n=2), ST15 (n=1), ST2128-1LV (n=1), and ST54 (n=1), respectively, and belong to the IncFIB(k) **(Figures S4-S6)**. The plasmids pPLA_020097, pOXA1_095005, pYZ-58-173k, and pKp_04A025_1 carry various antibiotic resistance genes as described below: pPLA_020097 carries *tet*(A)-v1, *dfrA12*, *aadA2*, *sul1*, *sul2*, *floR*, *bla*$_{TEM-176}$*-like*, *qnrS1*, and *aph(3')-Ib* **(Figure S6E)**; pOXA1_095005 carries *tet*(A)-v1, *bla*$_{OXA-1}$, *dfrA14*, *qnrB1*, *dfrA12*, *aadA2*, *sul1*, *mph(A)*, and *sul2* **(Figure S5D)**; pYZ-58-173k carries *tet*(A)-v1, *sul2*, *dfrA12*, and *aadA2* **(Figure S6B)**; pKp_04A025_1 carries an extensive set of resistance genes including *sul2*, *floR*, *dfrA12*, *aadA2*, *cmlA1*, *sul3*, *aac(3)-IId*, *qnrS1*, and *bla*$_{TEM-1B}$ **(Figure S6C)**. pNDM33-1 aligns with ST307 (n=1) and is categorized under the IncHI2. p1-S1-KEN-04-A aligns with ST4424 (n=1) and belongs to the IncFII **(Figure S5C)**. The plasmid carries the following antibiotic resistance genes: *tet*(A)-v1, *cmlA*, *aadA1*, *aph(4)-Ia*, *aph(3)-Ia*, *bla*$_{NDM-5}$, *bla*$_{TEM-1B}$, *bla*$_{OXA-10}$, and *floR* **(Figures S5C)**. The presence of multiple resistance genes on these plasmids can confer multidrug resistance to the host bacteria, posing significant challenges in treating infections caused by such

pathogens.

Detailed information of the four isolates (BSIKP-28, terRKP-23, terRKP-228, and GJY-G6) subjected to long-read sequencing using the MinION sequencer is as follows. BSIKP-28 contains two plasmids and one chromosome. Among these, plasmid pBSIKP-28_ *tet*(A)-v1 is an IncFIB(κ) conjugative plasmid, with a length of 161,160 bp, encoding 175 open reading frames (ORFs), and a G+C content of 51.1%. It contains resistance genes such as *tet*(A)-v1, *dfrA*2, *sul*1, *sul*2, *qnrS*1, *strB*, and *strA* **(Figure S6E)**. This plasmid shares 90% coverage and 99.9% identity with plasmid pPLA2_020097 (GenBank accession: CP045991) in the NCBI database. terRKP-23 contains three large plasmids, one chromosome, and several smaller plasmids. Plasmid pterRKP-23_*tet*(A)-v1 is an IncA/C2 conjugative plasmid, with 148,580 bp in length, encoding 174 ORFs with a G+C content of 52%. It harbors resistance genes such as *tet*(A)-v1, *floR*, *sul*2, *bla*$_{CMY\_2}$, *cmlA*1, *aadA*1, *bla*$_{OXA-10}$, *strB*, and *strA*. This plasmid shares 90% coverage and 100% identity to the p205880-Ct1/2 plasmid (GenBank accession: MF344573) **(SFigure S4C)**. Based on the SNP analysis, GJY-G6 and terRKP-23 belong to the same clone **(Table S2)**. Compared to terRKP-23, GJY-G6 is slightly shorter, with a length of 125,260 bp, encoding 153 ORFs, and a G+C content of 52%. It contains resistance genes such as *tet*(A)-v1, *qnrS1*, *floR*, *sul2*, and *drfA14*, and aligns with the same plasmid sequence as p205880-ct12 in NCBI **(Figure S4C)**. terRKP-228 contains two plasmids and one chromosome. Plasmid pterRKP-228_*tet*(A)-v1 is an IncFII conjugative plasmid, 140,811 bp in length, encoding 158 ORFs, and with a G+C content of 54.8%. It contains resistance genes including *tet*(A)-v1, *aac(3)-IId, floR*, *sul2*, *sul*1,*aadA1*, *aadA5*, *dfrA17*, *mph*(A), and *bla*$_{NDM-5}$. It shares 79% coverage and 100% identity to plasmid p1-S1-KEN-04-A (GenBank accession: CP145691) **(Figure S6D)**.
